# Identification of New Antimicrobial Peptides from Mediterranean Medical Plant *Charybdis pancration* (Steinh.) Speta

**DOI:** 10.3390/antibiotics9110747

**Published:** 2020-10-28

**Authors:** Vincenzo Cunsolo, Rosario Schicchi, Marco Chiaramonte, Luigi Inguglia, Vincenzo Arizza, Maria Grazia Cusimano, Domenico Schillaci, Antonella Di Francesco, Rosaria Saletti, Fabrizio Lo Celso, Giampaolo Barone, Maria Vitale

**Affiliations:** 1 Department of Chemical Sciences, University of Catania, Viale A. Doria 6, 95125 Catania, Italy; vcunsolo@unict.it (V.C.); antonelladfrancesco@gmail.com (A.D.F.); rsaletti@unict.it (R.S.); 2 Department of Agricultural Food and Forest Sciences (SAAF), University of Palermo, 90128 Palermo, Italy; rosario.schicchi@unipa.it; 3 Department of Biological, Chemical and Pharmaceutical Sciences and Technologies (STEBICF), University of Palermo, 90123 Palermo, Italy; marco.chiaramonte01@unipa.it (M.C.); luigi.inguglia@unipa.it (L.I.); vincenzo.arizza@unipa.it (V.A.); mariagrazia.cusimano@unipa.it (M.G.C.); giampaolo.barone@unipa.it (G.B.); 4 Department of Physics and Chemistry (DFC), University of Palermo, 90128 Palermo, Italy; fabrizio.locelso@unipa.it; 5 Ionic Liquids Laboratory, Institute of Structure of Matter, Italian National Research Council (ISM-CNR), 00133 Rome, Italy; 6 Istituto Zooprofilattico Sperimentale della Sicilia “A. Mirri”, 90129 Palermo, Italy; maria.vitale@izssicilia.it

**Keywords:** *Charybdis pancration* (Steinh.) Speta, antimicrobial peptides from plants, plant defensins, temporins, antibiotic resistant strains, high-resolution mass spectrometry, molecular dynamics

## Abstract

The present work was designed to identify and characterize novel antimicrobial peptides (AMPs) from *Charybdis pancration* (Steinh.) Speta, previously named *Urginea maritima*, is a Mediterranean plant, well-known for its biological properties in traditional medicine. Polypeptide-enriched extracts from different parts of the plant (roots, leaves and bulb), never studied before, were tested against two relevant pathogens, *Staphylococcus aureus* and *Pseudomonas aeruginosa*. With the aim of identifying novel natural AMPs, peptide fraction displaying antimicrobial activity (the bulb) that showed minimum inhibitory concentration (MICs) equal to 30 µg/mL against the above mentioned strains, was analysed by high-resolution mass spectrometry and database search. Seventeen peptides, related to seven proteins present in the investigated database, were described. Furthermore, we focused on three peptides, which due to their net positive charge, have a better chance to be AMPs and they were investigated by molecular modelling approaches, in order to shed light on the solution properties of their equilibrium structures. Some of new detected peptides could represent a good platform for the development of new antimicrobials in the fight against antibiotic resistance phenomenon.

## 1. Introduction

To control the spread of antibiotic resistance (AMR) new molecules worldwide to treat infectious diseases in patients are urgently required. Therapeutic intervention through conventional antibiotics are increasingly ineffective due to the selection of resistant pathogens. The threat for AMR is so widely spread in the environment, and in many fields of human activity, that all international health organizations have recently suggested a multidisciplinary, ONE-Health approach to control it. Resistant strains are present and eventually selected in fields, such as agriculture, animal health, food production and of course in hospital settings [1].

For this reason alternative antimicrobials are also needed to fight the AMR phenomenon in clinical settings, such as in agriculture, farms and food premises, to reduce or replace the use of most antibiotics, as well as last resort antibiotics used in human medicine to fight infectious diseases caused by otherwise resistant strains [2].

Antimicrobial peptides (AMPs) are encouraging molecules to substitute or to use in synergy with classical antibiotics to face the AMR. The synergic action can avoid, in any case, an over use of the antibiotics rendering more effective a low dosage therapy and lowering the selection of resistant strains. AMPs represent the first line of defense against pathogens in almost all life forms, from prokaryotes to humans [3,4]. AMPs are molecules made up of a variable number of amino acids between 10 and 50, endowed with a wide spectrum of biological activities that leads them to act, not only as antimicrobials, but also as anti-biofilm, anti-proliferative, anti-inflammatory agents and immune modulators [5,6]. Their antibacterial activity is reached through different mechanisms, such as targeting bacterial membranes, including the lipopolysaccharides layer, and interacting with intracellular targets such as DNA, RNA and the protein synthesis machinery [7,8].

Many active antimicrobial peptides have been analyzed from many different sources from prokaryotes, to fungi and yeast to marine organisms and plants. The extreme biodiversity of the plants makes them a recognized source of novel AMPs, compared to other organisms. There is of scientific interest in relation to plants due to their considerable variability, both in amino acid sequence and in secondary and tertiary structures, together with several activities as potent antimicrobial, antifungal, antiprotozoal and antiviral [9]. The first AMP identified in plants dates back to the 1990s, and it was a defensin isolated from wheat and barley seeds. In more recent years, hundreds of defensins with antibacterial, antifungal and insecticidal activity have been found in family plant as *Arabidopsis* and *Medicago* [10]. Most of AMPs from plants are endowed in particular amino acids, such as cysteine, and consequently of various disulfide bonds (usually two to six), which give a more organized and resistant secondary structure [11]. Plant defensins are classified according to the number of cysteines into 8C-plant defensins with four disulfides and 10C-plant defensins with five disulfide bonds [12]. Other peptides such as cyclic peptides, cyclotides, show many biological activities including antimicrobial properties [13,14]. Antimicrobial activity of cyclotides was first described for some cyclotides derivatives from coffee plants, kalata B1, circulin A, circulin B, and cyclopsychotride [15].

In this study, we focused, in particular, on the research of novel peptides from the *Charybdis pancration* (Steinh.) Speta plant, previously named *Uringea maritima* (L.) Baker, family plant *Asparagaceae* Juss. Such plant, also called squill, is well-known in traditional medicine from Mediterranean region to Persia, and it has been used in many medical treatments, such as bacterial and helminthic infections, rheumatism, edema, gout, abortion induction, wound healing, etc. [16]. Due to their content in glycosides, flavonoids and phenolic compounds [16,17,18,19,20,21] *C. pancration* extracts are known for their potential use in the treatment of many diseases, especially respiratory and heart diseases. Furthermore, among glycosides produced, the scilliroside has a potent rodenticidal activity [22], which affects cardiovascular and CNS, causing convulsions and death.

Contrary to the previous studies on this medical plant [23], we have analyzed and characterized the amino acidic fraction of the *C. pancration* extracts which, to the best of our knowledge, has never been studied before for its potential AMPs content. Roots, leaves and bulb of the plant were separately collected and subjected to acid extraction in order to obtain the protein fraction. Then, the polypeptide-enriched extracts were tested for their antimicrobial activity against two important pathogens, *Staphylococcus aureus* and *Pseudomonas aeruginosa*, which are common antibiotic resistant strains frequently responsible of polymicrobial infections in chronic wounds in nosocomial setting and veterinary medicine [24]. The fraction displaying an antimicrobial activity (the bulb) was further analyzed by High-Pressure Liquid Chromatography/nano-Electrospray Ionization tandem Mass Spectrometry (RP-HPLC/nESI-MS/MS) with the aim of identifying and characterizing the polypeptide active extract’s content and seventeen novel peptides sequences were described. Molecular dynamics (MD) simulations were performed on three of identified potential AMPs with the aim of defining their conformation.

## 2. Results

### 2.1. C. pancration Peptide Extraction and Antimicrobial Activity

**Figure 1:**
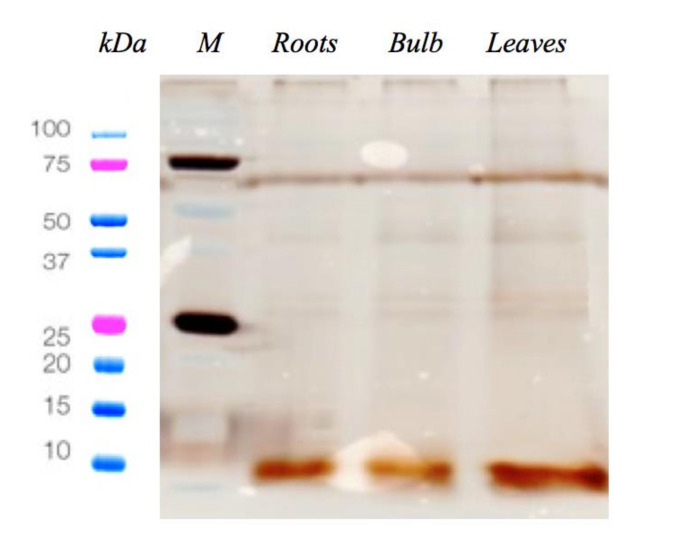
Silver staining of total acid extract from *C. pancration* after SDS- PAGE. (Line M, Molecular Weight Standards; line Roots, Proteins extracted from test plant roots; line Bulb, Proteins extracted from test plant bulb; line Leaves, Proteins extracted from test plant leaves).

Cationic peptides were extracted from *C. pancration* roots, bulb and leaves and a sodium dodecyl sulfate polyacrylamide gel electrophoresis (SDS- PAGE) assay was performed to check the extraction process (Figure 1).

The antimicrobial activity of the three polypeptide-enriched extracts obtained from roots, leaves and bulb of *C. pancration* (Steinh.) Speta were evaluated starting from a 50% *v/v* concentration of each sample, against two reference bacterial strains, *S. aureus* ATCC 25923 and *P.aeruginosa* ATCC 15442.

The results obtained were included in Table 1 and are expressed in terms of Minimum inhibitory concentration (MIC) with the values reported in percentage v/v and in concentration µg/mL of protein content. The most interesting sample is the one taken from the bulb that showed a MIC of 12.5% *v/v*, corresponding to 30.5 µg/mL of protein content, against both pathogens, instead the other two samples from roots and leaves were inactive at the maximum tested concentration of 50% *v/v* corresponding respectively to a protein concentration of 20 or 45 µg/mL.

In the light of these results, only the bulb sample extract was investigated further. In particular, with the goal of characterizing the amino acid structures of peptides, a RP-HPLC/nESI-MS/MS analysis was performed.

### 2.2. MS Characterization of the Amino Acid Sequence of the Peptides Present in the Bulb Extract

With the aim of identifying the peptide components and characterizing their amino acid sequences, the bulb extract, the only fraction displaying an antimicrobial activity, was analyzed by RP-HPLC/nESI-MS/MS. Database search allowed the characterization of seventeen peptides (Table 2; see Supplementary Materials S1 for more details). Sixteen out of seventeen peptide sequences were related to seven *Viridiplantae*-derived proteins, whereas one quality peptide sequence, detected by de novo sequencing, cannot be traced back to proteins present in the database investigated. When the same set of peptides identified more proteins from different species that could not be differentiated based on MS/MS analysis alone, they were grouped to satisfy the principles of parsimony (groups of parsimony). These proteins are reported in Table 2 with the annotation multispecies identification. Then, in order to predict the potential of the molecules identified as AMPs and similarities with already described AMPs, the “APD3: Antimicrobial Peptide Calculator and Predictor” tool of the Antimicrobial Peptide Database (APD) has been used [25].

In detail, eight peptide sequences were related to the Ribosome-inactivating protein charybdin of *C. pancration* (Steinh.) Speta. Peptide #1 (ILDISYNKNALQD) shows 46.7% of amino acid similarity with hylain 1 AMP of frog (*Hyla simplex*) [26]. Peptide sequence #2 (SEPVKLPQWMQND) presents 44.4% of similarity with hemerycin, a nematode AMP, from the polychaete *Marphysa sanguinea* [27], having potent activity against Gram-negative and Gram-positive bacteria. Peptide # 3

(VDIANHFAFN) may interact with membranes. It has a similarity of 37.5% to temporins, a family of short (8–17 amino acids), hydrophobic peptides with antibacterial and antifungal properties that are synthesized in the skins of a wide range of North American and Eurasian frogs of the *Ranidae* family [28]. Similarly, Peptide #5 shows 35.6% amino acid similarity with temporin AMP. Finally, also the peptide sequences #4, # 6, # 7, and # 8 present amino acid similarity with AMPs produced by other organisms including prokaryotes [29] (see Table 2). They may adopt α-helical conformation in hydrophobic environments, show some hydrophobic residues, and potentially have the ability to perturb the integrity of bacterial cell membranes. Consequently, these peptides might present a potential antimicrobial activity.

Two out of the seventeen peptide sequences (e.g., #9, VVTFGPTGLTTEVK and #10, IERSTNLDWYKGPTLL) derive from the Elongation factor 1-alpha. In detail, the peptide VVTFGPTGLTTEVK shows 41.2% amino acid similarity with bacteriocin, a peptidic toxins produced by the bacterium *Pediococcus pentosaceus* and able to inhibit the growth of similar or closely related bacterial strain(s). The second peptide, which may have a α-helical conformation, instead shows 38.9% similarity with a frog-derived AMP temporin.

Analogously, other two peptide sequences #11 and #12 (QIPLTGAHSIIGRA and IPLSGPNAVIGRA) show 40% similarity with a frog AMP temporin Rb. They are related to the proteins Superoxide dismutase and Superoxide dismutase of chloroplast respectively, show some hydrophobic residues and could interact with the bacterial cell membranes and therefore represent potential antimicrobial peptides.

Two peptide sequences #13 (LHTFRLPPFL) and #14 (LEELLLHT) derive from the protein Allene oxide synthase 4. The first one shows 40% similarity with the frog AMP temporin-1Gc, whereas the peptide #14 has 50% similarity with the gageotetrin C, a not ribosomally synthesized peptide antibiotic from *Bacillus subtilis*. However, it cannot be predicted whether or not these two amino acid sequences are capable to form a α-helix so long to represent antimicrobial peptides.

Finally, also the three peptides #15, #16 and #17 present amino acid similarity with frog-derived AMP temporins. In particular, peptide LSRSMKEAGFKLDW correspond with the trait LLRSMKEAGFKLDW of a RTM3-like protein, but carrying the amino acid substitution Ser→Leu (reported as underlined). This sequence has 41.2% similarity with the frog Temporin-1La, but we do not know if it is able to form a α-helix so long to be an antimicrobial peptide. The peptide VSLPINELLD, related to the Photosystem I P700 apoprotein chlorophyll, represents another potential AMP because shows 50% amino acid similarity with the frog temporin E, and due to its potential ability to form a α-helix and interact with the bacterial membrane.

The last peptide (e.g., #17, FVCPLNLLAE) represent the unique sequence identified in the bulb extracts that cannot be directly traced back to known proteins of the investigated database. Therefore, a BLAST (Basic Local Aligment Search Tool; https://blast.ncbi.nlm.nih.gov/Blast.cgi) database search of peptide #17 was carried out. BLAST search, which permits to find regions of similarity between biological sequences, allowed to ascertain that the amino acid sequence FVCPLNLLAE has only two amino acid differences with the trait FVCPLSLQAE of an uncharacterized protein (NCBI seq. Id XP_024392827.1) from *Physcomitrella patens*. The sequence FVCPLNLLAE might adopt α-helical conformation in hydrophobic environments, has a similarity of 42.8% with the frog-derived AMP temporin-Ra, and therefore could represent a potential antimicrobial agent.

Among the detected molecules, we focused on the three peptides #6, #11 and #12, due to their net positive charge, which potentially provides a better chance of targeting the bacterial membrane. In fact, it has been reported that cationic peptides more often act as good active AMPs, since they interact better with the anionic bacterial cell membranes. Principal physicochemical parameters were obtained by using in silico analysis, giving insight into their potential mechanism of action using the Antimicrobial Peptide Database [25] and Half-Life of Peptides (HLP), a useful tool to predict the half-life of peptides in a biological proteolytic environment [30]. As an example, the Boman index showed that peptide #6 (LEKNWVRFSF) has a higher affinity for proteins, instead the peptides #11(QIPLTGAHSIIGRA) and #12 (IPLSGPNAVIGRA) are hydrophobic peptides displaying high values of Wimley–White whole residue hydrophobicity scales and consequently they have a better possibility of interacting and perturbing the bacterial membrane, which is the main target of action of many AMPs. Other interesting parameters regarding peptides #11 and #12 are the values of half-life and high stability, see Table 3.

### 2.3. Molecular Dynamics Simulations

**Figure 2:**
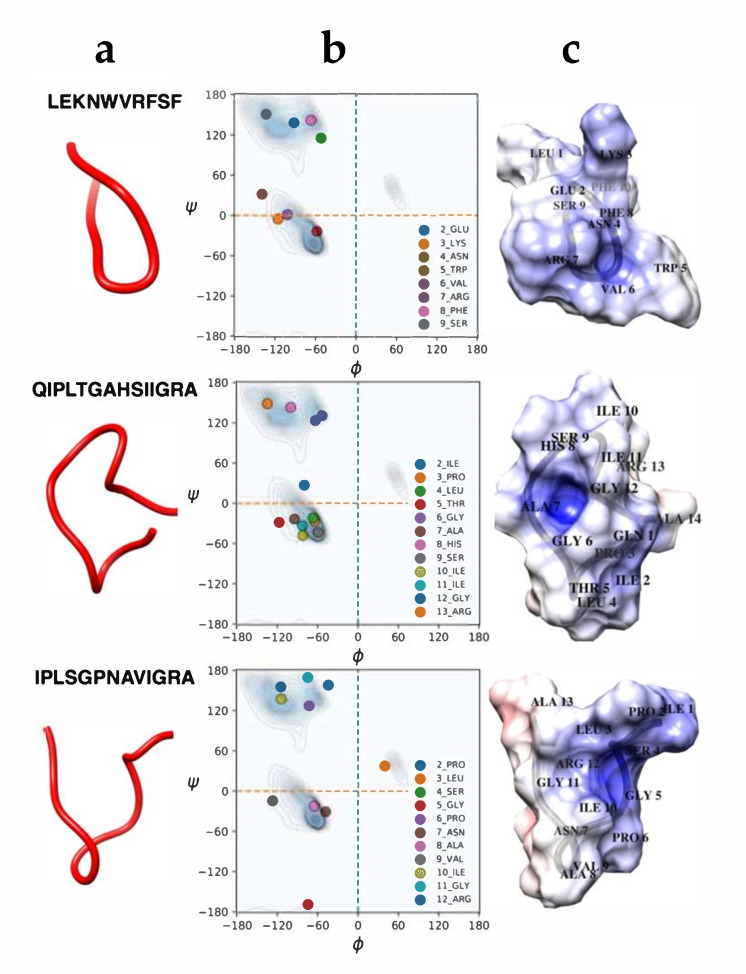
(**a**) Most representative equilibrium structures of peptides #6, #11 and #12, obtained by MD simulations. (**b**) Ramachandran plots showing the values of the psi (Ψ) and phi (Φ) angles assumed by each residue, as indicated. (**c**) Electrostatic potential of the peptides, shown as surfaces; red, blue and white colors are used for negative (−3 kT/e), positive (+7 kT/e), and zero values, respectively.

In order to deepen our knowledge on the characteristics of peptides #6, #11 and #12, we used molecular dynamics models, 1 µs MD simulations were performed on the three peptides. The most representative equilibrium structures, shown in Figure 2, attest that they essentially assume random coil conformations. Moreover, the calculated electrostatic potential shows that they are all characterized by a positive (blue) region in a pocket formed during the simulation, near the VAL6, ALA7, GLY 5 residues for peptides #6, 11 and #12, respectively. Interestingly, the three peptides show also protruding regions, located near LYS 3, HIS 8 and ILE1 residues, for peptides #6, #11 and #12, respectively. Such protruding regions are generally associated with a hydrophobic region able to interact with the fatty acid side chains of the lipids, that can potentially interact with the bacterial membrane. Moreover, the intensity of the red and blue colors, higher for peptide #11 and #12, compared to that of peptide #6, witness the slightly hydrophobicity of peptide #6, in agreement with the Wimley–White data discussed above.

## 3. Discussion

Due to AMR spreading worldwide, there is a pressing demand for broad-spectrum antimicrobial agents, especially against drug-resistant Gram-negative pathogens [31]. In such a scenario, it is necessary the development of new and unconventional anti-infective therapies. In this regard, AMPs have been of particular interest, mainly for their different mechanisms of action compared to the most common antibiotics. Novel antimicrobials are particularly needed also towards *S. aureus* and *P. aeruginosa,* included on the WHO/OMS priority bacteria list for their high or critical antibiotic resistance [32]. The two microorganisms have ample diffusion in natural environments, but also in nosocomial setting and in veterinary practices. They are the most common causes of polymicrobial infectious diseases, as corneal infection or wound associated infections (diabetic foot, venous leg and pressure ulcers) [33], and are also involved in animal health and food safety issues [17].

In this paper, we focused on the amino acidic fraction of the bulb of the medical plant *C. pancration* which showed an interesting activity against *S. aureus* and *P. aeruginosa*. The RP-HPLC/nESI-MS/MS analysis (Table 2), has revealed the presence of sixteen peptide sequences related to seven Viridiplantae-derived proteins. The detected peptides share a great percentage of similarity in the sequence with known AMPs, but it is interesting to observe that they are different respect to AMPs from plants so far described. The major families of AMPs from plants are characterized by the presence of multiple disulfide bonds, whereas we found that novel peptides from *C. pancration* are not rich in cysteine residues.

Moreover, twelve out of seventeen peptides are largely similar with AMPs, present in amphibians, and in particular, with temporins that exhibit an antimicrobial activity against Gram positive, Gram negative pathogens and fungi [34]. Among all the peptides identified, two of them, the peptides #11 and #12, showed high similarity with frog derived temporin A and temporin B, that recently has been indicated as good template to design in vivo active antimicrobials [35]. We believe that the peptides #11 and #12 are also good candidate as AMPs themselves, but also as chemical platforms to develop effective antimicrobial drugs due to their physicochemical parameters, such as their net positive charge, the presence of hydrophobic residues and their stability, that render them potentially able to approach and interact with bacterial membrane. However, to better understand the role of the above peptides from *C. pancration*, their antibacterial response mechanisms and toxicity toward eucaryotic cells further studies will be performed on synthetic homologous and some derivate peptides.

## 4. Materials and Methods

### 4.1. Plant Collection and Plant Extract Preparation

The plant was collected in Madonie mountains (Sicily) and identified by one of the authors, R.S. (Rosario Schicchi), director of the Botanical Garden at the University of Palermo (Italy). Plants were cleaned and divided in different samples (roots, leaves, bulb). Subsequently, they were cut with a sterile scalpel, homogenized and kept at −80 °C until following analyses. Cationic peptides were extracted according to well-established protocols [14,36,37,38,39]. In brief, roots, leaves and bulb freeze-dried samples (1 g/sample) were dissolved in 3 mL of extraction buffer (10% acetic acid in phosphate saline buffer) and sonicated for 60 s at 0 °C (setting: 1 pulse/s, 70% duty cycle). The samples were then centrifuged at 27,000× *g* at 4 °C for 30 min, supernatants were collected, freeze-dried and re-dissolved in sterile water for further analysis.

### 4.2. Protein Content Determination

The protein concentration of the sample aliquots was measured utilizing the Qubit Protein Assay Kit through the Qubit 2.0 fluorometer (ThermoFisher, Waltham, MA, USA), according to the manufacturer’s instructions. The protein pattern was resolved in a 12% Sodium Dodecyl Sulfate (SDS) polyacrylamide gel electrophoresis according to the method described [37], followed by visualization with silver staining.

### 4.3. Bacterial Strains

Two reference strains have been used *S. aureus* ATCC 25923 and *P.aeruginosa* ATCC 15442. The media used in this study were tryptic soy broth (TSB, Sigma-Aldrich, Merck Life Science S.r.l., Milano, Italy) and tryptic soy agar (TSA).

### 4.4. Determination of Minimal Inhibitory Concentrations (MICs)

MICs of plant extracts were determined by a broth dilution micro-method using Tryptic Soy Broth (TSB) in 96 wells plate by diluting 1:2 the extracts starting from a 50% *v/v* in a final volume per well equal to 100 μL [38]. Briefly, a bacterial suspension, obtained by diluting a bacterial culture grown at 37 °C for 24 h on Tryptic Soy Agar (TSA) in 5 mL of 0.9% NaCl, whose turbidity was equivalent to a 0.5 McFarland standard and viability count of 5 × 10^6^ CFU/mL, was added (10 μL per well) into the wells of 96-wells plate. MICs of extracts were recorded as the lowest concentration of sample whose optical density (OD) at 570 nm (Glomax Multidetection System Promega, Milano, Italy) is comparable to negative control (broth with extract without bacterial inoculum). A positive growth control (tested strain in the medium without any antibiotic molecule), a sample control (only the sample solution without inoculum, to control the absorbance of samples at the tested concentrations) and a negative control (only the medium without inoculum as a control of the sterility of medium), were also added in the test. Each assay was performed in triplicates and repeated at least twice.

### 4.5. Mass Spectrometry Analysis

The characterization of the peptides, present in the bulb extract, was performed by nLC-nESI MS/MS using a Thermo Scientific Dionex UltiMate 3000 RSLCnano system (Thermo Fisher Scientific, Sunnyvale, CA, USA) coupled on-line with a Thermo Fisher Scientific Orbitrap Fusion Tribrid^®^ (Q-OT-qIT) mass spectrometer (Thermo Fisher Scientific, Bremen, Germany). The solution of the bulb extract was subjected to filtration through a 0.45 µm filter (Alltech, Lexington, KY, USA). Then, 1 µL of the solution was loaded onto a trapping column (Acclaim^®^ Nano Trap C18 Column; 2 cm × 100 µm ID, 5 µm, 100 Å) and washed with solvent A (H_2_O/ACN, 95/5 + 0.1% FA) at a flow rate of 7 µL/min for 3 min. Then, the solution was switched from the trapping column onto a PepMap^®^ RSLC C18 EASY-Spray column (50 cm × 75 µm ID, 2 µm, 100 Å). Chromatographic peptide separation was performed at a flow rate of 0.25 µL/min and 40 °C using a modified method previously adopted [40]. In particular, peptides were separated with a linear gradient of solvent B (ACN + 0.1% FA) in A from 5% to 40% in 32 min, followed by 40% to 60% in 10 min, and by 60% to 95% in other 5 min. We finished by holding 95% B for 5 min, by 95% to 5% in 10 min, and finally re-equilibrating the column at 5% B for 20 min. Peptide cations were converted to gas-phase ions by electrospray ionization using the following parameters: (i) source voltage, 1.9 kV; (ii) ion transfer tube temperature, 275 °C. Precursor peptides were detected from 400 to 1600 m/z with a resolution of 120 K (@ 200 m/z) using the following parameters: RF lens, 60%; Auto Gain Control target, 400,000; maximum injection time, 50 ms. Then, MS/MS data were acquired for up to 3 s and a maximum parallel injection time of 250 ms per precursor mass. Precursor ions were isolated in the quadrupole with 1.6 Th and a 10 ppm tolerance around the selected precursor and its isotopes. Then, they were fragmented using a normalized collision energy of 35, following the automatic exclusion for 60 s. Only those precursors with charge state 1÷4 and an intensity above the threshold of 5 × 10^3^ were sampled for MS/MS. Peptide mixtures were analysed running a MS technical duplicate per sample. Mass spectrometer calibration was performed by using the Pierce^®^ LTQ Velos ESI Positive Ion Calibration Solution (Thermo Fisher Scientific, Bremen, Germany). MS data acquisition was carried out by utilizing the Xcalibur v. 3.0.63 software (Thermo Fisher Scientific, Bremen, Germany).

### 4.6. Database Search

MS/MS data were analyzed and searched by the PEAKS de novo sequencing software (v. 7.0, Bioinformatics Solutions Inc., Waterloo, ON, Canada) using a modified approach previously adopted [40,41,42]. In particular, de novo amino acid sequences, generated from each spectrum, were searched against the UniProt protein sequences database limited to *Viridiplantae* (green plants) taxonomy (April 2017 release). The following parameters were used: i) Enzymes: none; ii) variable modifications: oxidation of methionine, transformation of N-terminal glutamine and N-terminal glutamic acid residue in the pyroglutamic acid form, deamidation of aspartic or glutamic acid, acetylation of lysine, phosphorylation of serine, threonine or tyrosine. The precursor mass tolerance threshold was 10 ppm and the max fragment mass error was set to 0.6 Da. Peptide spectral matches (PSM) were validated using a Target Decoy PSM Validator node based on q-values at a 1% FDR. All Peptides Spectrum Matches were also manually checked.

### 4.7. AMP Prediction and in Silico Analysis

To identify principal physicochemical characteristics of some found peptides, such as hydrophobicity, affinity to attach to other proteins (Boman index), isoelectric point etc., we employed the “APD3: Antimicrobial Peptide Calculator and Predictor” tool of the Antimicrobial Peptide Database (APD) [25]. The predicted half-life and stability of the new peptides in an intestine-like, proteolytic environment was determined with the aid of “HLP: Web server for predicting half-life of peptides in intestine-like environment” [30].

### 4.8. Molecular Dynamics Simulations

Molecular dynamic (MD) simulations were performed using the GROMACS 2019.4 package [39,43,44]. The simulations for the various systems were performed using a cubic box of explicit TIP3P water [45], with a distance between the walls and the polypeptide atoms of 10 Å.

Periodic boundary conditions were applied. The Amber ff99SB force field [44] was used and parameter files and initial configuration for the polypeptides were created by GROMACS utilities programs. The equilibration procedure was done in several steps, starting from a 2 ns simulation at 300 K, at constant values of number of particles, volume and temperature (NVT ensemble), with the polypeptide heavy atom positions restrained to equilibrate the solvent around it, followed by a run at 300 K and pressure at 1 bar, at constant values of number of particles, pressure and temperature (NPT ensemble), for 2 ns. After the equilibration phase, the system was run for a total of 1000 ns for a NVT production run; the trajectory was saved at a frequency of 10 ps. The simulations were always checked against the root mean square displacement (RMSD) and the energy profile. During the production runs for the temperature coupling, a velocity rescaling thermostat [46] (with a time coupling constant of 0.1 ps) was used, selecting a Parrinello–Rahman barostat [47] for the pressure coupling (1 ps for the relaxation constant). The Leap-Frog algorithm with a 2 fs time step was used for integrating the equations of motion. Cut-offs for the Lennard-Jones and real space part of the Coulombic interactions were set to 10 Å. For the electrostatic interactions, the Particle Mesh Ewald (PME) summation method [48,49] was used, with an interpolation order of 4 and 0.16 nm of Fast Fourier Transform (FFT) grid spacing. Selected images and polypeptide manipulation were done using Chimera program [50,51,52].

Ramachandran contour lines were considered for the General case (non-Gly, non-Pro, and non-prePro) [53]. Electrostatic potential surfaces were obtained by the PDB2QR [54] and APBS [55] procedures.

## 5. Conclusions

In conclusion, the detected activity on the extracts from the natural source (the bulb of the plant) represents a good starting point to considerer the relevant peptides as a good candidate platform to develop new antimicrobials in clinical settings to overcome antibiotic resistance in infectious diseases, but also in applicative processes in non-clinical settings, such as food safety and agriculture.

## Figures and Tables

**Table 1:** Minimum inhibitory concentration (MIC) of the three samples from different parts of *C. pancration.*

Strains	MIC		
	Roots	Leaves	Bulb
*S.aureus* ATCC 25923	>50% *v/v* (>20 µg/mL)	>50% *v/v* (>45 µg/mL)	12.5% *v/v* (30.5 µg/mL)
*P.aeruginosa* ATCC 15442	>50% *v/v* (>20 µg/mL)	>50% *v/v* (>45 µg/mL)	12.5% *v/v* (30.5µg/mL)

Activity expressed as MIC in % *v/v* or µg/ mL of protein content in brackets.

**Table 2:** Proteins, related peptides and some characteristics as potential AMPs from *C. pancration*.

Protein (Acc. No; Taxa)	#No.	Identified Peptides	Predicted Ability to Interact with MembranesBetter Chance to Be an AMP	Similarity with already Described AMPs
Ribosome-inactivating protein charybdin (*P84786; C.pancration*)				
	#1	ILDISYNKNALQD	-	46.7% frog AMP (hylain1)
	#2	SEPVKLPQWMQND	-	44.4% nematode AMP
	#3	VDIANHFAFN	yes	37.5% frog AMP (temporin)
	#4	ILDISYNKNALQDAVSK	yes	45.4% frog AMP (cruzioseptin)
	#5	LPQWMQNDLEKN	-	35.7% AMP frog (temporin)
	#6	LEKNWVRFSF	yes	40% AMP prokaryotes
	#7	VDIANHFAFNLE	yes	41.7% AMP frog (hylain 1)
	#8	DILDISYNKNALQD	yes	46.6% AMP frog (hylain 1)
Elongation factor 1-alpha (*multispecies identification*)				
	#9	VVTFGPTGLTTEVK	-	41.2% bacteriocin (*Pediococcus pentosaceus*)
	#10	IERSTNLDWYKGPTLL	yes	38.9% frog AMP (temporin)
Superoxide dismutase [Cu-Zn] (*multispecies identification*)				
	#11	QIPLTGAHSIIGRA	yes	40% AMP frog (temporin Rb)
Superoxide dismutase [Cu-Zn], chloroplastic (*multispecies identification*)				
	#12	IPLSGPNAVIGRA	yes	40% AMP frog (temporin Rb)
Allene oxide synthase (*multispecies identification*)				
	#13	LHTFRLPPFL	-	40% AMP frog (temporin-1Gc)
	#14	LEELLLHT	-	50% gageotetrin (*Bacillus subtilis*)
RTM3-like protein (*multispecies identification*)				
	#15	LSRSMKEAGFKLDW	-	41.2% AMP frog (temporin 1La)
Photosystem P700 chlorophyll (*multispecies identification*)				
	#16	VSLPINELLD	yes	50% AMP frog (temporin E)
Unknown				
	#17	FVCPLNLLAE	yes	42.8% AMP frog (Temporin-Ra)

**Table 3:** Principal physicochemical parameters of the three potential AMPs #6, #11 and #12.

**Chemical-Physical Properties**	**Peptide #6**	**Peptide #11**	**Peptide #12**
Peptide sequence	LEKNWVRFSF	QIPLTGAHSIIGRA	IPLSGPNAVIGRA
Theoretical mass (Da)	1325.67	1433.89	1264.68
Net Charge	+1	+1	+1
Isoelectric point	6.360	6.401	6.336
			
Wimley-White whole-residue hydrophobicity (kcal/mol)	−0.23	1.15	1.51
			
Protein-binding potential Boman index (kcal/mol)	2	0.42	0.05
Half-life (s)	0.0001	1.754	1.531
Stability	Low	High	High

## Supplementary Materials

The following are available online at https://www.mdpi.com/2079-6382/9/11/747/s1, S1: Mass Spectrometry Analysis.
