# Supplementary material for: Identification of New Antimicrobial Peptides from Mediterranean Medical Plant Charybdis pancration (Steinh.) Speta
Source: Antibiotics (Basel). 2020 Oct 28;9(11):747. doi: 10.3390/antibiotics9110747 (PMC7694139; doi:10.3390/antibiotics9110747)
Supplement: Supplementary file 1 [file antibiotics-09-00747-s001.pdf]

Supplementary Materials

S1: Mass Spectrometry Analysis.

N.	Description Protein (Acc. No.; *Taxa*)	N.	Peptide	Score (-10lgP)	Mass	AA Length	ppm	m/z	z	RT (min)	Scan
1	Ribosome-inactivating protein charybdin (P84786; *C.pancration*)	#1	ILDISYNKNALQD	61.82	1505.7726	13	1	753.8943	2	32.03	F1:7037
2		#2	SEPVKLPQWMQND	71.62	1570.745	13	0.7	786.3803	2	34.24	F2:10073
3		#3	VDIANHFAFN	67.31	1146.5458	10	0.6	574.2805	2	33.28	F2:9627
4		#4	ILDISYNKNALQDAVSK	65.74	1891.005	17	0.5	631.3426	3	31.43	F2:8731
5		#5	LPQWMQNDLEKN	60.46	1514.7188	12	−0.1	758.3666	2	33.24	F1:7607
6		#6	LEKNWVRFSF	57.24	1324.6927	10	0	663.3536	2	33.26	F1:7615
7		#7	VDIANHFAFNLE	57.6	1388.6724	12	0.2	695.3436	2	37.02	F2:11377
8		#8	DILDISYNKNALQD	46.72	1620.7994	14	0.3	811.4072	2	35.88	F1:8795
9	Elongation factor 1-alpha (*Multisepcies identification*)	#9	VVTFGPTGLTTEVK	67.89	1447.7922	14	−0.3	724.9032	2	33.09	F2:9535
10		#10	IERSTNLDWYKGPTLL	56.9	1904.9995	16	−0.3	636.0069	3	35.05	F2:10458
11	Superoxide dismutase [Cu-Zn] 2 (*Multisepcies identification*)	#11	QIPLTGAHSIIGRA	54.47	1432.8151	14	1	478.6128	3	28.96	F2:7531
12	Superoxide dismutase [Cu-Zn], chloroplastic (*Multisepcies identification*)	#12	IPLSGPNAVIGRA	57.98	1263.7299	13	−0.4	632.8719	2	30.69	F1:6408
13	Allene oxide synthase 4 (*Multisepcies identification*)	#13	LHTFRLPPFL	37.94	1239.7128	10	0	620.8636	2	36.34	F2:11040
14		#14	LEELLLHT	41.29	966.5386	8	−0.3	484.2764	2	33.41	F1:7684
15	RTM3−like protein (*Multisepcies identification*)	#15	LSRSMKEAGFKLDW	42.22	1666.8501	14	−3.8	834.4291	2	32.22	F1:7130
16	Photosystem P700 chlorophyll (*Multisepcies identification*)	#16	VSLPINELLD	41.81	1111.6124	10	0.1	556.8135	2	36.15	F1:8917
17	Unknown (De novo sequence)	#17	FVCPLNLLAE	92% [a)]	1117.5842	10	−3.4	559.7975	2	36.09	F2:8889

a) Amino acid sequence, derived by de novo analysis, that cannot be directly traced back to known proteins of the investigated database. The score Reported refers to the Average Local Confidence (ALC %). PEAKS assigns a local confidence score for each amino acid in a de novo sequence. The local confidence score ranges from 0% to 99%, indicating how confident the algorithm considers a particular amino acid as the correct assignment. So that, ALC is the average of the local confidence score of all the amino acids in the sequence.

Ribosome-inactivating protein charybdin (*P84786; C.pancration*)

#1 ILDISYNKNALQD

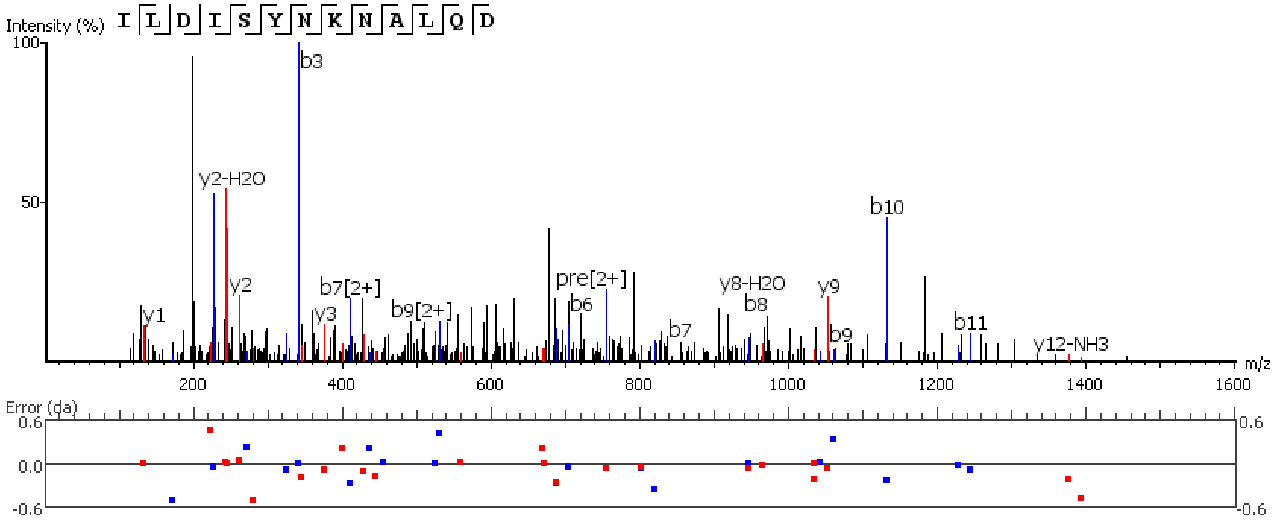

#	b	b-H2O	b-NH3	b (2+)	Seq	y	y-H2O	y-NH3	y (2+)	#
1	114.09	96.08	97.06	57.55	I					13
2	227.22	209.17	210.15	114.09	L	1394.18	1375.69	1376.88	697.35	12
3	342.21	324.29	325.18	172.13	D	1280.61	1262.60	1263.58	640.81	11
4	455.26	437.28	438.04	228.14	I	1165.58	1147.57	1148.56	583.29	10
5	542.32	524.30	525.29	271.42	S	1052.57	1034.70	1035.45	526.75	9
6	705.45	687.37	688.63	353.19	Y	965.50	947.53	948.44	483.23	8
7	819.79	801.41	802.47	410.49	N	802.47	784.39	785.38	401.48	7
8	947.53	929.51	930.49	474.26	K	688.63	670.14	671.33	344.88	6
9	1061.22	1043.53	1044.54	530.84	N	560.23	542.26	543.24	281.14	5
10	1132.84	1114.59	1115.57	566.80	A	446.41	428.21	429.32	223.14	4
11	1245.79	1227.67	1228.70	623.34	L	375.28	357.18	358.16	188.09	3
12	1373.74	1355.73	1356.72	687.37	Q	262.04	244.06	245.06	131.55	2
13					D	134.06	116.03	117.02	67.52	1

Ribosome-inactivating protein charybdin (*P84786; C.pancration*)

#2 SEPVKLPQWMQND

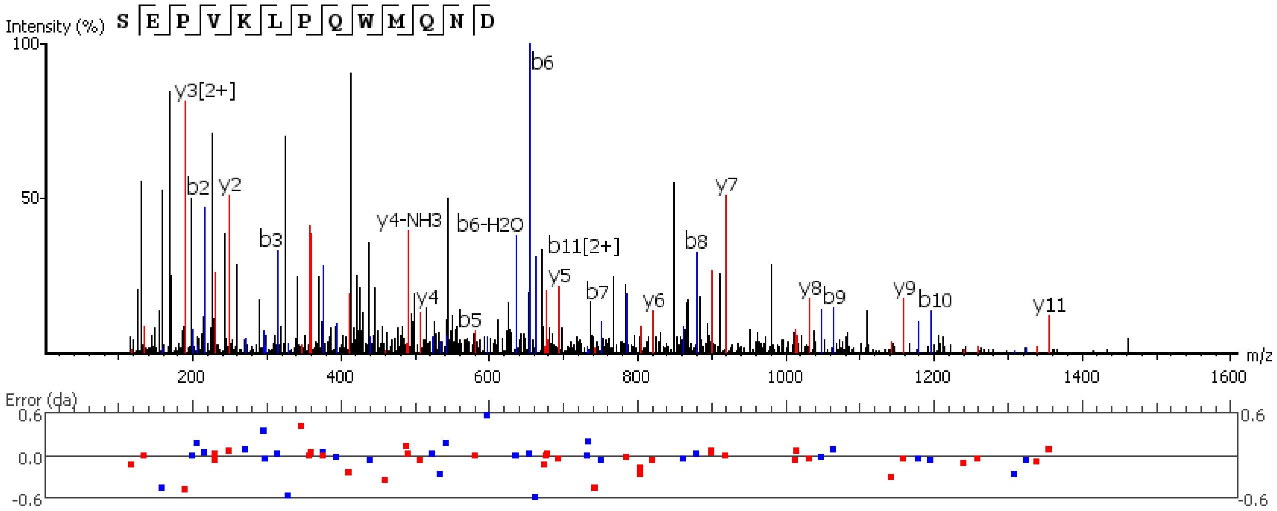

#	b	b-H2O	b-NH3	b (2+)	Seq	y	y-H2O	y-NH3	y (2+)	#
1	88.04	70.03	71.01	44.52	S					13
2	217.02	199.05	200.06	109.04	E	1484.72	1466.71	1467.69	743.32	12
3	314.11	295.77	297.16	158.03	P	1355.59	1337.67	1338.74	678.32	11
4	413.20	395.22	396.18	206.93	V	1258.68	1240.72	1241.60	629.81	10
5	541.12	523.26	524.27	271.06	K	1159.61	1141.85	1142.84	580.29	9
6	654.35	636.36	637.36	328.28	L	1031.51	1013.53	1014.36	516.23	8
7	751.50	733.42	734.21	376.17	P	918.38	900.31	901.28	460.04	7
8	879.46	861.54	862.47	440.33	Q	821.41	803.58	804.48	411.42	6
9	1065.48	1047.60	1048.55	533.55	W	693.32	675.39	676.24	346.72	5
10	1196.69	1178.66	1179.59	598.23	M	507.27	489.04	490.12	254.09	4
11	1324.76	1306.66	1307.91	663.43	Q	376.17	358.14	359.06	189.06	3
12	1438.72	1420.70	1421.69	719.86	N	248.01	230.16	231.02	124.54	2
13					D	134.07	116.03	117.16	67.52	1

Ribosome-inactivating protein charybdin (*P84786; C.pancration*)

#3 VDIANHFAFN

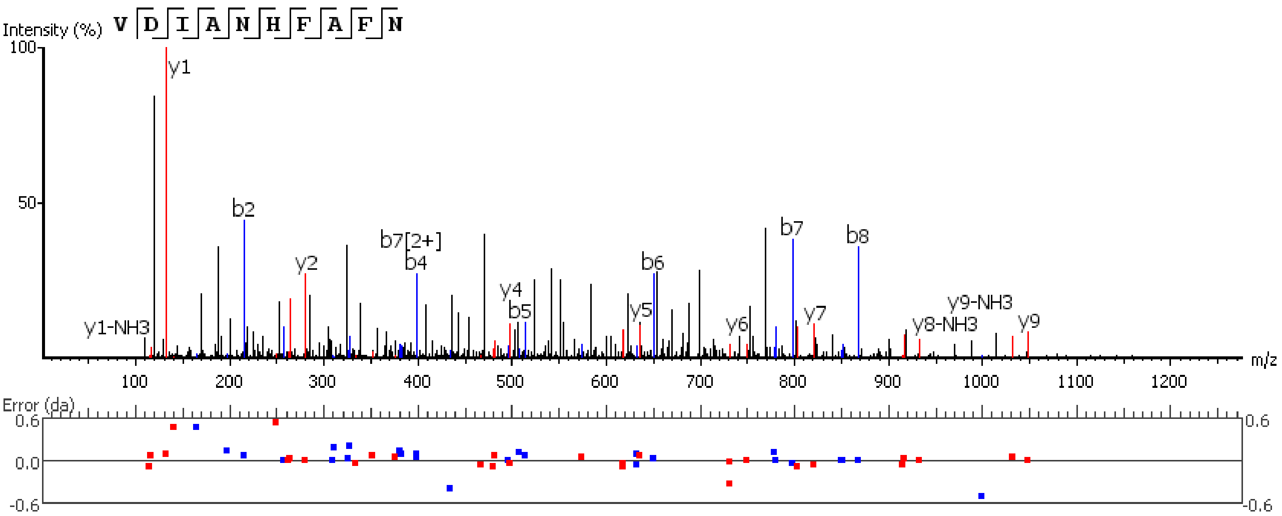

#	b	b-H2O	b-NH3	b (2+)	Seq	y	y-H2O	y-NH3	y (2+)	#
1	100.08	82.07	83.05	50.54	V					10
2	215.03	196.95	198.08	108.05	D	1048.49	1030.47	1031.41	524.74	9
3	327.98	310.16	310.98	164.12	I	933.47	915.52	916.40	467.30	8
4	399.13	381.07	382.10	200.11	A	820.45	802.36	803.45	410.69	7
5	513.19	495.26	496.25	257.13	N	749.35	731.67	732.35	375.12	6
6	650.28	632.40	633.19	325.64	H	635.21	617.35	618.37	318.15	5
7	797.46	779.27	780.38	399.13	F	498.29	480.33	481.12	249.07	4
8	868.42	850.41	851.40	435.13	A	351.09	333.16	334.19	176.08	3
9	1015.50	997.49	999.01	508.13	F	280.14	262.13	263.07	140.09	2
10					N	132.97	115.15	115.97	67.03	1

Ribosome-inactivating protein charybdin (*P84786; C.pancration*)

#4 ILDISYNKNALQDAVSK

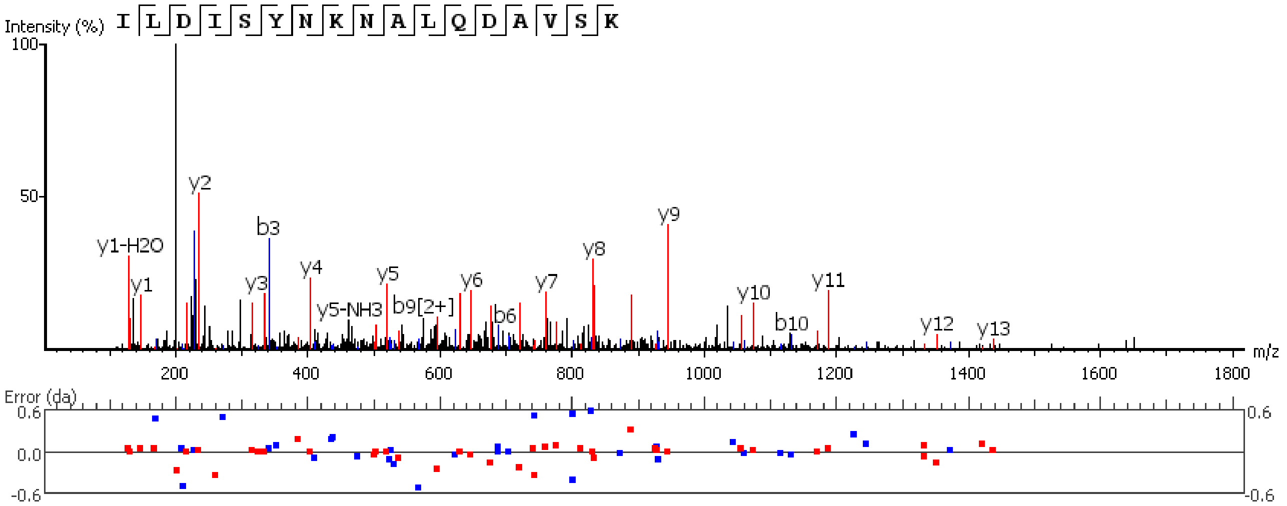

#	b	b-H2O	b-NH3	b (2+)	Seq	y	y-H2O	y-NH3	y (2+)	#
1	114.09	96.08	97.06	57.55	I					17
2	227.14	209.10	210.67	114.09	L	1778.93	1760.92	1761.90	889.63	16
3	342.15	324.18	325.18	171.12	D	1665.84	1647.83	1648.82	833.53	15
4	455.29	437.09	438.04	228.14	I	1550.82	1532.81	1533.79	775.80	14
5	542.32	524.42	525.25	271.15	S	1437.70	1419.72	1420.58	719.61	13
6	705.38	687.37	688.27	353.09	Y	1350.86	1332.59	1333.76	676.03	12
7	819.43	801.84	801.84	410.31	N	1187.59	1169.63	1170.61	594.59	11
8	947.52	929.43	930.63	474.33	K	1073.57	1055.58	1056.51	537.40	10
9	1061.60	1043.41	1044.54	531.47	N	945.49	927.43	928.45	473.25	9
10	1132.66	1114.59	1115.62	567.34	A	831.44	813.38	814.43	416.23	8
11	1245.56	1227.41	1228.66	623.39	L	760.33	742.36	743.75	380.71	7
12	1373.72	1355.73	1356.72	687.37	Q	647.39	629.32	630.31	324.18	6
13	1488.77	1470.76	1471.74	744.36	D	519.27	501.32	502.27	260.49	5
14	1559.81	1541.80	1542.78	780.40	A	404.25	386.06	387.22	202.90	4
15	1658.88	1640.86	1641.85	829.34	V	333.19	315.17	316.14	167.06	3
16	1745.91	1727.90	1728.88	873.48	S	234.10	216.15	217.12	117.57	2
17					K	147.06	129.04	130.11	74.06	1

Ribosome-inactivating protein charybdin (*P84786; C.pancration*)

#5 LPQWMQNDLEKN

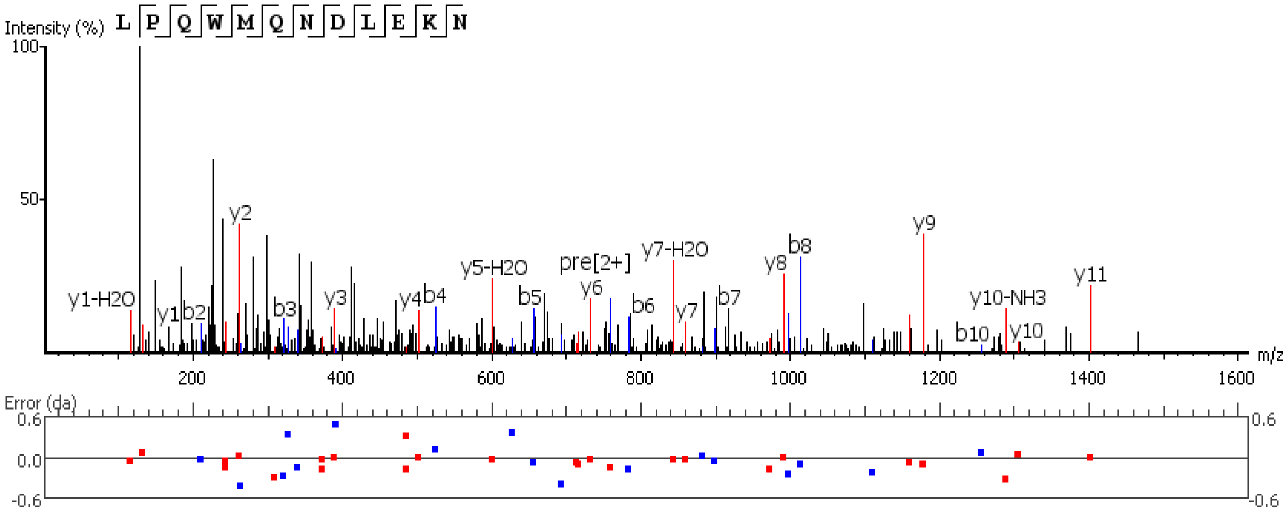

#	b	b-H2O	b-NH3	b (2+)	Seq	y	y-H2O	y-NH3	y (2+)	#
1	114.09	96.08	97.06	57.55	L					12
2	211.19	193.13	194.12	106.07	P	1402.63	1384.63	1385.61	701.82	11
3	339.35	321.48	322.18	170.10	Q	1305.52	1287.58	1288.90	653.29	10
4	525.14	507.27	508.26	263.57	W	1177.65	1159.62	1160.50	589.27	9
5	656.40	638.31	639.30	328.30	M	991.45	973.63	974.42	496.23	8
6	784.56	766.37	767.35	392.20	Q	860.45	842.43	843.38	430.71	7
7	898.48	880.41	881.35	449.71	N	732.38	714.42	715.44	366.68	6
8	1013.57	995.44	996.69	507.23	D	618.31	600.33	601.28	309.97	5
9	1126.54	1108.53	1109.73	563.77	L	503.29	484.93	486.44	252.14	4
10	1255.50	1237.57	1238.55	627.91	E	390.17	372.38	373.21	195.60	3
11	1383.67	1365.66	1366.65	692.74	K	261.11	243.21	244.29	131.08	2
12					N	132.99	115.11	116.03	67.03	1

Ribosome-inactivating protein charybdin (*P84786; C.pancration*)

#6 LEKNWVRFSF

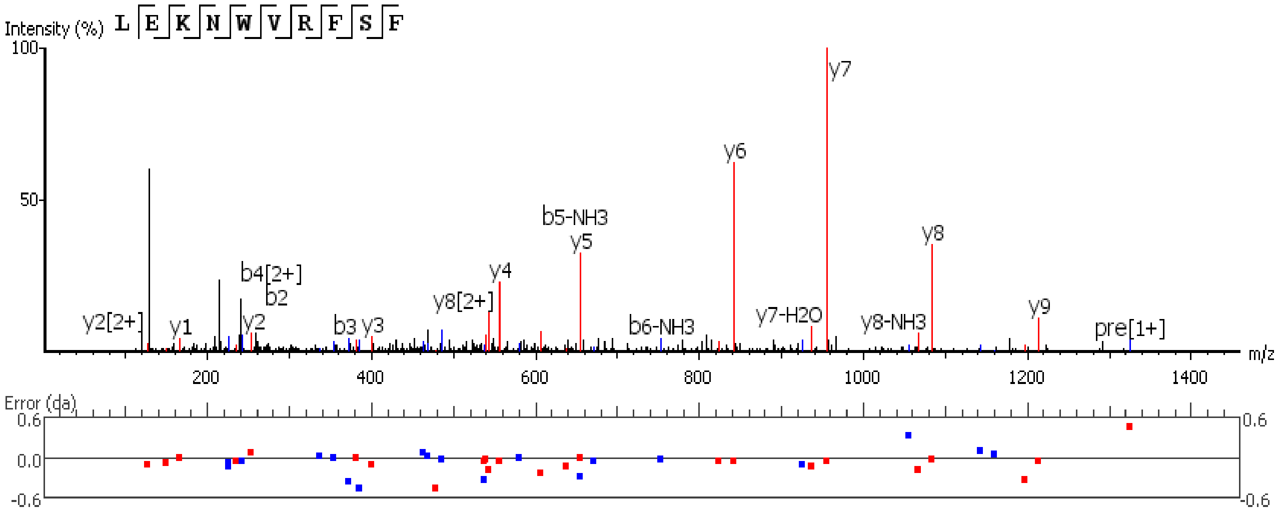

#	b	b-H2O	b-NH3	b (2+)	Seq	y	y-H2O	y-NH3	y (2+)	#
1	114.09	96.08	97.06	57.55	L					10
2	243.20	225.21	226.25	122.07	E	1212.68	1194.61	1195.93	607.06	9
3	371.60	353.23	354.20	186.11	K	1083.62	1065.56	1066.75	542.48	8
4	485.30	467.26	468.22	243.20	N	955.53	937.61	938.45	478.70	7
5	671.41	653.34	654.61	336.14	W	841.49	823.42	824.46	421.22	6
6	770.42	752.41	753.44	386.17	V	655.36	637.35	638.47	328.18	5
7	926.63	908.51	909.49	463.66	R	556.34	538.34	539.29	278.64	4
8	1073.59	1055.58	1056.23	537.63	F	400.30	382.19	383.16	200.59	3
9	1160.55	1142.50	1143.59	580.79	S	253.04	235.18	236.09	127.18	2
10					F	166.11	148.08	149.14	83.54	1

Ribosome-inactivating protein charybdin (*P84786; C.pancration*)

#7 VDIANHFAFNLE

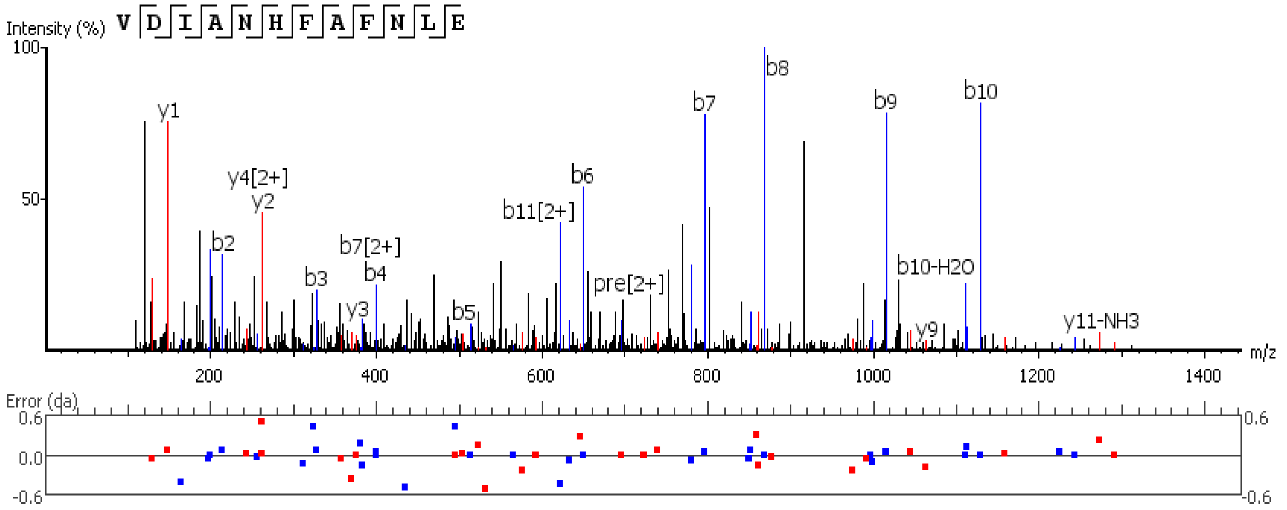

#	b	b-H2O	b-NH3	b (2+)	Seq	y	y-H2O	y-NH3	y (2+)	#
1	100.08	82.07	83.05	50.54	V					12
2	215.03	197.16	198.08	108.05	D	1290.62	1272.60	1273.35	645.51	11
3	328.10	310.18	311.31	165.01	I	1175.58	1157.57	1158.53	588.29	10
4	399.17	381.03	382.35	200.13	A	1062.69	1044.49	1045.41	532.27	9
5	513.26	494.80	496.23	257.16	N	991.52	973.45	974.69	496.23	8
6	650.35	632.41	633.30	325.21	H	877.45	859.10	860.56	439.21	7
7	797.32	779.46	780.37	399.17	F	740.27	722.35	723.34	371.05	6
8	868.41	850.49	851.31	435.21	A	593.32	575.28	576.51	297.15	5
9	1015.44	997.47	998.59	508.25	F	522.08	504.25	505.20	261.12	4
10	1129.56	1111.54	1112.38	565.25	N	375.21	357.18	358.24	188.09	3
11	1242.64	1224.62	1225.54	622.25	L	261.12	243.10	244.12	131.07	2
12					E	147.96	130.11	131.03	74.53	1

Ribosome-inactivating protein charybdin (*P84786; C.pancration*)

#8 DILDISYNKNALQD

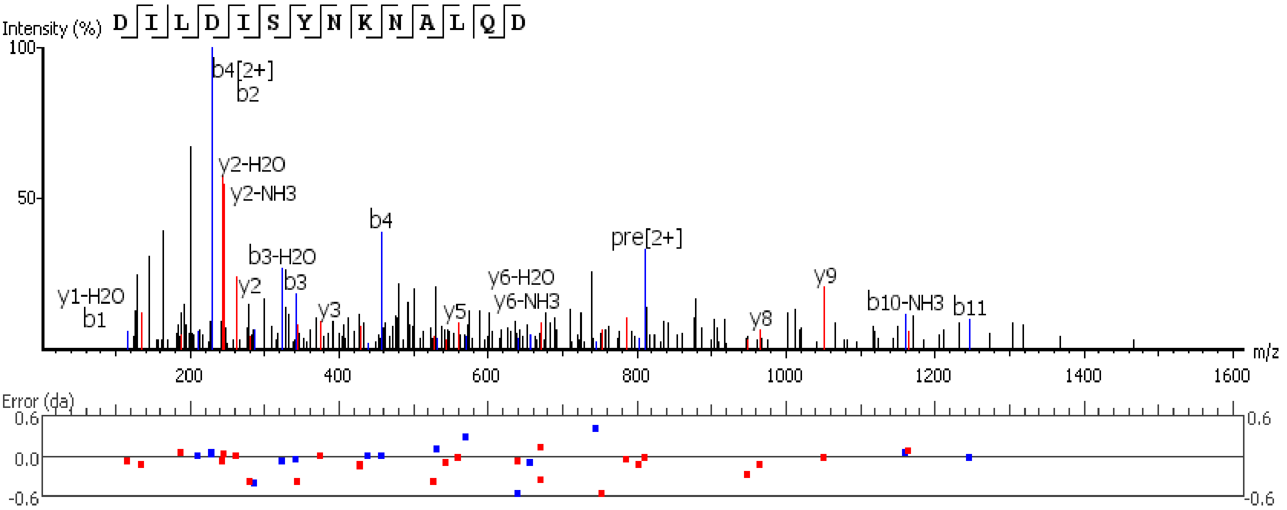

#	b	b-H2O	b-NH3	b (2+)	Seq	y	y-H2O	y-NH3	y (2+)	#
1	116.12	98.02	99.01	58.52	D					14
2	229.07	211.12	212.09	115.06	I	1506.78	1488.77	1489.75	754.46	13
3	342.27	324.28	325.18	171.60	L	1393.70	1375.69	1376.67	697.35	12
4	457.21	439.21	440.20	229.07	D	1280.61	1262.60	1263.58	640.91	11
5	570.02	552.30	553.29	286.08	I	1165.50	1147.57	1148.56	583.29	10
6	657.46	639.34	640.91	329.17	S	1052.55	1034.49	1035.47	527.14	9
7	820.41	802.53	803.38	410.70	Y	965.62	947.46	948.73	483.23	8
8	934.45	916.44	917.43	467.73	N	802.53	784.39	785.44	401.70	7
9	1062.55	1044.54	1045.52	531.67	K	688.36	670.20	671.69	345.09	6
10	1176.59	1158.58	1159.51	588.80	N	560.31	542.26	543.35	281.02	5
11	1247.68	1229.62	1230.60	624.31	A	446.22	428.36	429.36	223.61	4
12	1360.71	1342.70	1343.68	680.86	L	375.17	357.18	358.16	188.04	3
13	1488.77	1470.76	1471.74	744.45	Q	262.08	244.18	245.05	131.55	2
14					D	134.18	116.12	117.02	67.52	1

Elongation factor 1-alpha (*multispecies identification*)

#9 VVTFGPTGLTTEVK

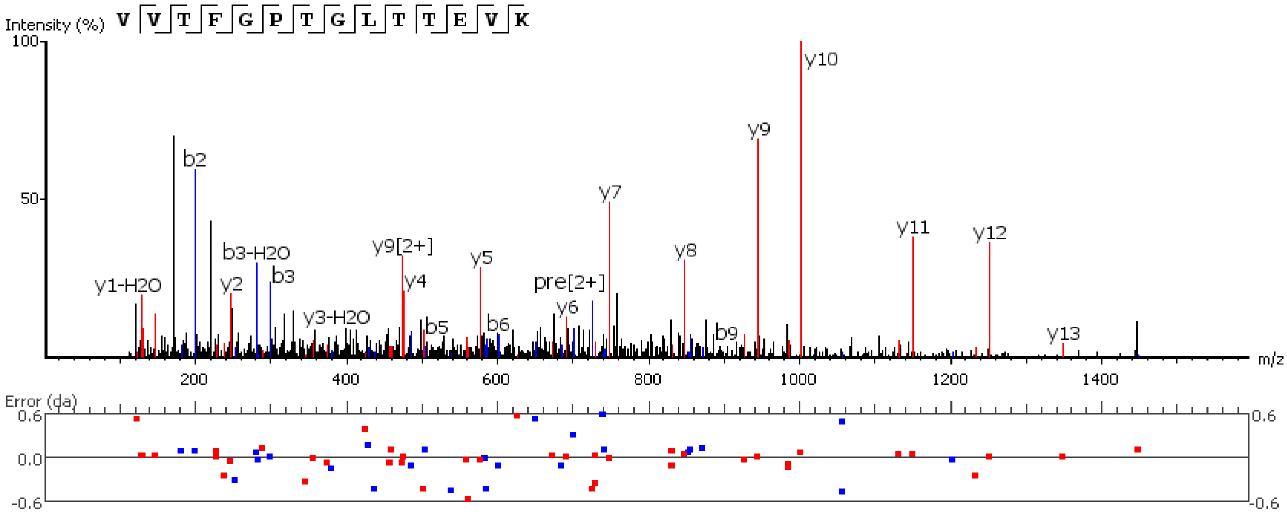

#	b	b-H2O	b-NH3	b (2+)	Seq	y	y-H2O	y-NH3	y (2+)	#
1	100.08	82.07	83.05	50.54	V					14
2	199.06	181.04	182.12	100.07	V	1349.74	1331.72	1332.70	675.37	13
3	300.20	282.11	283.22	150.60	T	1250.68	1232.92	1233.64	625.26	12
4	447.26	429.09	430.23	224.13	F	1149.57	1131.55	1132.59	575.31	11
5	504.17	486.39	487.26	252.97	G	1002.48	984.68	985.63	502.23	10
6	601.46	583.36	584.76	301.17	P	945.51	927.57	928.50	473.35	9
7	702.07	684.49	685.36	351.69	T	848.42	830.37	831.57	424.35	8
8	759.40	740.80	742.28	380.38	G	747.45	729.39	730.77	374.30	7
9	872.36	854.41	855.36	437.19	L	690.42	672.37	673.38	346.05	6
10	973.54	955.53	956.51	487.27	T	577.38	559.36	560.88	289.04	5
11	1074.58	1057.06	1057.06	538.26	T	476.29	458.35	459.14	238.90	4
12	1203.68	1185.62	1186.60	602.31	E	375.22	357.24	358.20	188.11	3
13	1302.69	1284.68	1285.67	651.32	V	246.24	228.18	229.07	123.05	2
14					K	147.09	129.07	130.06	74.06	1

Elongation factor 1-alpha (*multispecies identification*)

#10 IERSTNLDWYKGPTLL

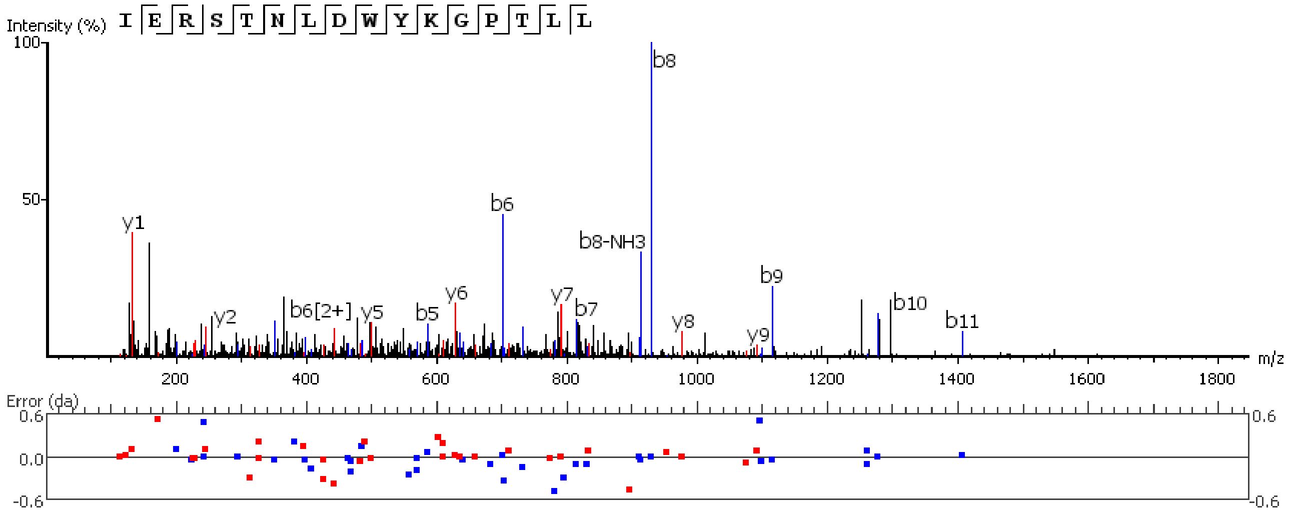

#	b	b-H2O	b-NH3	b (2+)	Seq	y	y-H2O	y-NH3	y (2+)	#
1	114.09	96.08	97.06	57.55	I					16
2	243.13	225.17	226.11	122.07	E	1792.92	1774.91	1775.90	897.43	15
3	399.29	381.01	382.21	200.01	R	1663.88	1645.87	1646.85	832.34	14
4	486.11	468.48	469.32	243.13	S	1507.78	1489.77	1490.75	754.39	13
5	587.25	569.50	570.33	294.18	T	1420.75	1402.74	1403.72	710.78	12
6	701.33	683.46	684.33	351.24	N	1319.70	1301.69	1302.67	660.34	11
7	814.55	796.75	797.42	407.89	L	1205.66	1187.65	1188.63	603.04	10
8	929.46	911.48	912.51	465.26	D	1092.48	1074.67	1075.55	546.79	9
9	1115.60	1097.01	1098.60	558.53	W	977.56	959.53	960.52	489.06	8
10	1278.61	1260.72	1261.48	639.86	Y	791.48	773.49	774.44	396.08	7
11	1406.68	1388.70	1389.68	704.20	K	628.38	610.39	611.17	315.01	6
12	1463.73	1445.72	1446.70	732.52	G	500.35	482.37	483.28	250.65	5
13	1560.78	1542.77	1543.75	781.39	P	443.67	425.60	426.31	222.14	4
14	1661.83	1643.82	1644.80	831.52	T	346.23	328.26	329.00	173.09	3
15	1774.91	1756.90	1757.89	887.96	L	245.08	227.22	228.20	123.06	2
16					L	131.99	114.09	115.08	66.55	1

Superoxide dismutase [Cu-Zn] (*multispecies identification*)

#11 QIPLTGAHSIIGRA

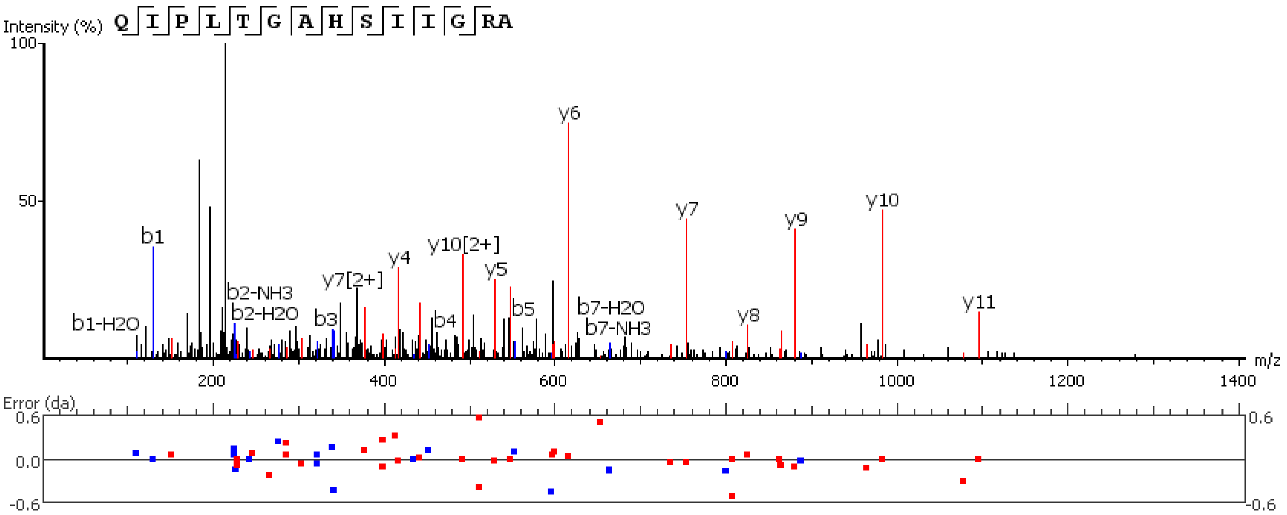

#	b	b-H2O	b-NH3	b (2+)	Seq	y	y-H2O	y-NH3	y (2+)	#
1	129.07	110.96	112.04	65.03	Q					14
2	242.13	224.06	224.98	121.58	I	1305.76	1287.75	1288.74	652.87	13
3	339.04	321.12	322.24	170.10	P	1192.68	1174.67	1175.65	596.77	12
4	452.16	434.28	435.24	226.79	L	1095.65	1077.94	1078.60	548.31	11
5	553.22	535.32	536.31	276.92	T	982.54	964.66	965.52	491.77	10
6	610.36	592.35	593.33	305.68	G	881.61	863.48	864.56	441.22	9
7	681.39	663.53	664.54	341.63	A	824.41	806.46	807.97	412.41	8
8	818.45	800.61	801.43	409.73	H	753.48	735.43	736.46	377.09	7
9	905.48	887.47	888.49	453.24	S	616.33	598.37	599.25	308.69	6
10	1018.57	1000.56	1001.54	509.78	I	529.37	511.73	511.73	265.41	5
11	1131.65	1113.64	1114.63	566.33	I	416.30	397.99	399.35	208.63	4
12	1188.67	1170.66	1171.65	595.30	G	303.26	285.09	285.91	152.02	3
13	1344.78	1326.76	1327.75	672.89	R	246.06	228.15	229.23	123.58	2
14					A	90.05	72.04	73.03	45.53	1

Superoxide dismutase [Cu-Zn], chloroplastic (*multispecies identification*)

#12 IPLSGPNAVIGRA

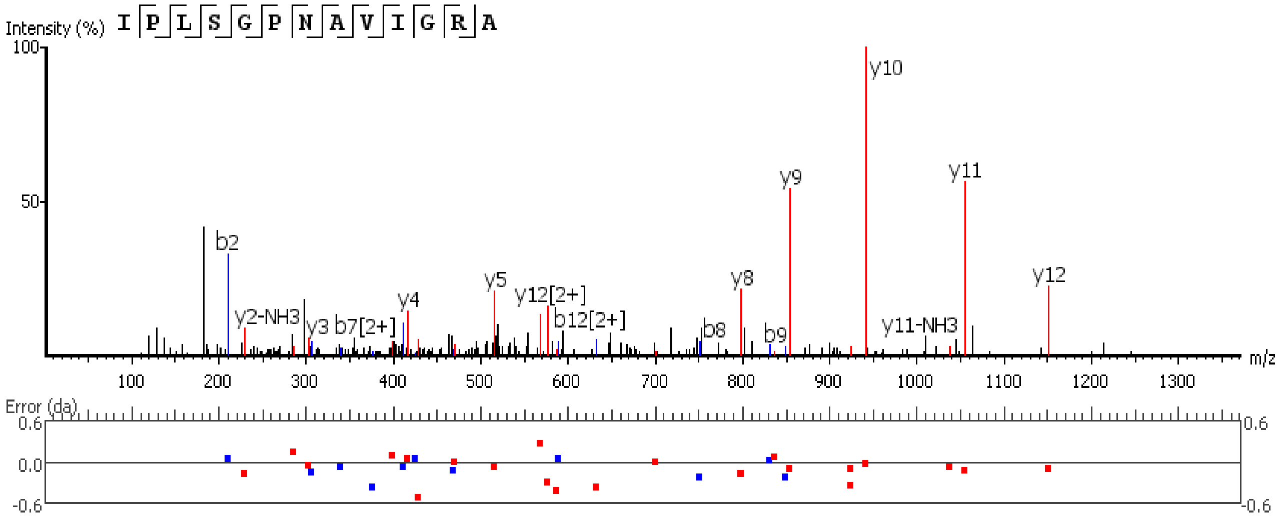

#	b	b-H2O	b-NH3	b (2+)	Seq	y	y-H2O	y-NH3	y (2+)	#
1	114.09	96.08	97.06	57.55	I					13
2	211.09	193.13	194.12	106.07	P	1151.77	1133.64	1134.63	576.63	12
3	324.23	306.38	307.20	162.61	L	1054.73	1036.59	1037.67	527.80	11
4	411.35	393.25	394.23	206.13	S	941.55	923.85	924.61	471.24	10
5	468.41	450.27	451.26	234.64	G	854.59	836.47	837.37	428.27	9
6	565.33	547.32	548.31	283.17	P	797.65	779.45	780.44	399.13	8
7	679.38	661.37	662.35	340.28	N	700.42	682.40	683.38	350.71	7
8	750.64	732.40	733.39	376.09	A	586.79	568.07	569.34	293.68	6
9	849.73	831.45	832.46	425.18	V	515.42	497.32	498.30	258.16	5
10	962.57	944.56	945.54	481.78	I	416.20	398.25	399.13	208.63	4
11	1019.59	1001.58	1002.56	510.29	G	303.23	285.17	285.99	152.09	3
12	1175.69	1157.68	1158.66	588.28	R	246.16	228.15	229.31	123.58	2
13					A	90.05	72.04	73.03	45.53	1

Allene oxide synthase *(multispecies identification*)

#13 LHTFRLPPFL

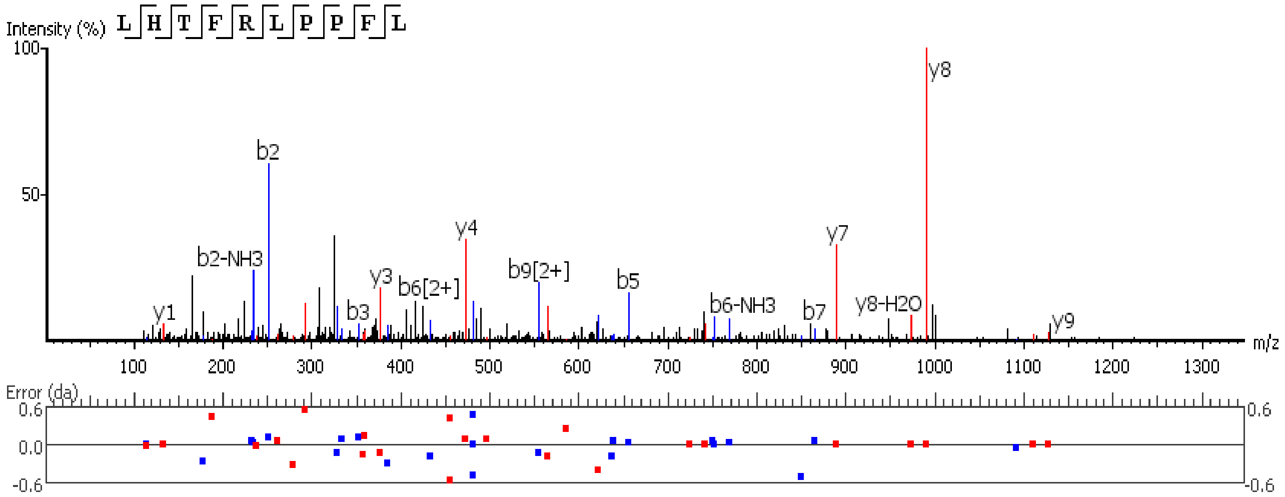

#	b	b-H2O	b-NH3	b (2+)	Seq	y	y-H2O	y-NH3	y (2+)	#
1	114.12	96.08	97.06	57.55	L					10
2	251.02	233.08	234.08	126.08	H	1127.61	1109.63	1110.60	564.52	9
3	352.08	334.10	335.17	176.89	T	990.56	972.59	973.55	495.70	8
4	499.27	481.77	481.77	250.13	F	889.54	871.52	872.50	445.26	7
5	655.34	637.56	638.28	328.35	R	742.47	724.43	725.43	371.73	6
6	768.41	750.37	751.43	385.03	L	586.11	568.35	569.33	293.12	5
7	865.44	847.49	849.01	433.44	P	473.19	455.84	455.84	237.17	4
8	962.56	944.55	945.53	481.77	P	376.37	358.38	359.06	188.15	3
9	1109.63	1091.68	1092.60	555.47	F	279.51	261.10	262.14	140.09	2
10					L	132.08	114.12	115.07	66.55	1

Allene oxide synthase (*multispecies identification*)

#14 LEELLLHT

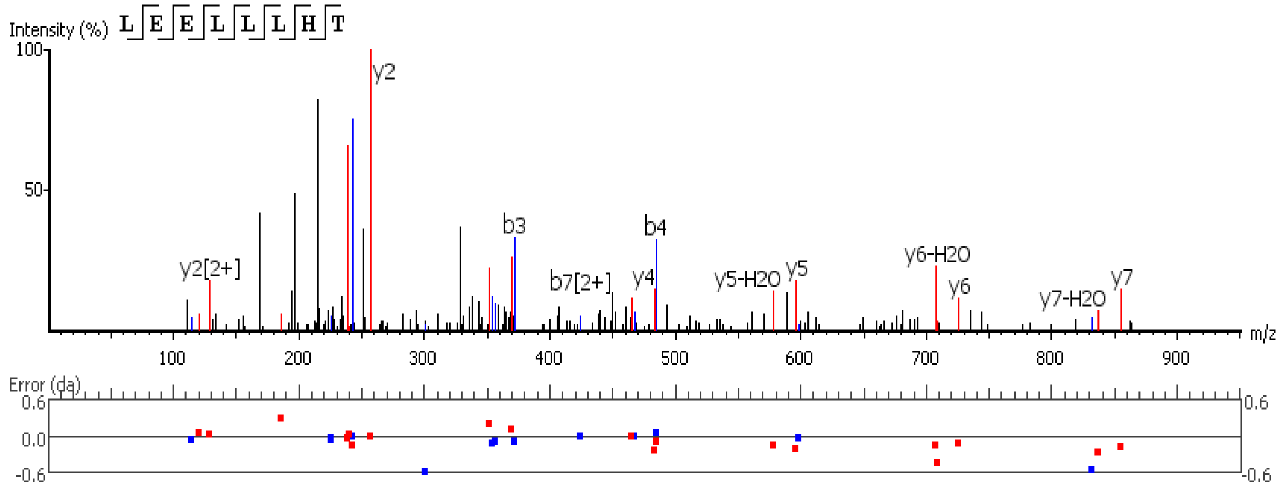

#	b	b-H2O	b-NH3	b (2+)	Seq	y	y-H2O	y-NH3	y (2+)	#
1	114.16	96.08	97.06	57.55	L					8
2	243.13	225.16	226.17	122.07	E	854.64	836.72	837.43	427.73	7
3	372.28	354.30	355.15	186.59	E	725.55	707.57	708.85	363.21	6
4	485.19	467.26	468.23	243.13	L	596.59	578.53	579.35	298.69	5
5	598.39	580.33	581.32	300.26	L	483.55	465.28	466.27	242.30	4
6	711.43	693.42	694.40	356.30	L	370.08	351.97	353.18	185.30	3
7	848.49	830.48	832.03	424.75	H	257.14	239.16	240.05	129.01	2
8					T	119.98	102.05	103.04	60.53	1

RTM3-like protein *(multispecies identification)*

#15 LSRSMKEAGFKLDW

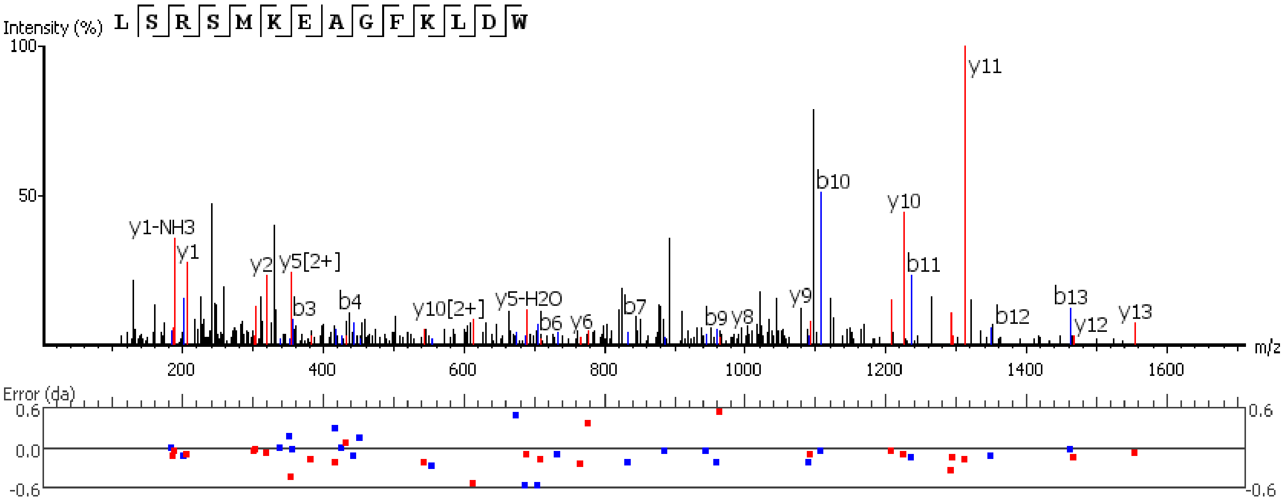

#	b	b-H2O	b-NH3	b (2+)	Seq	y	y-H2O	y-NH3	y (2+)	#
1	114.09	96.08	97.06	57.55	L					14
2	201.25	183.09	184.10	101.06	S	1554.85	1536.76	1537.75	777.52	13
3	357.25	339.20	340.20	179.11	R	1467.90	1449.73	1450.71	734.37	12
4	444.39	426.26	427.23	222.63	S	1311.81	1294.00	1294.78	656.32	11
5	575.30	557.29	558.27	288.15	M	1224.71	1206.60	1207.65	613.36	10
6	703.96	685.38	686.95	352.01	K	1093.68	1075.56	1076.54	547.28	9
7	832.66	814.42	815.41	416.42	E	964.93	947.46	948.45	483.24	8
8	903.47	885.52	886.45	452.08	A	836.43	818.42	819.40	418.72	7
9	960.73	942.48	943.54	480.75	G	765.64	747.38	748.37	383.37	6
10	1107.63	1089.55	1090.76	554.57	F	708.56	690.48	691.34	355.14	5
11	1235.83	1217.65	1218.63	618.33	K	561.30	543.29	544.50	281.15	4
12	1348.87	1330.73	1331.71	674.37	L	433.13	415.20	416.42	217.10	3
13	1463.81	1445.76	1446.74	732.49	D	320.22	302.17	303.14	160.56	2
14					W	205.20	187.22	188.13	103.05	1

Photosystem P700 chlorophyll *(multispecies identification)*

#16 VSLPINELLD

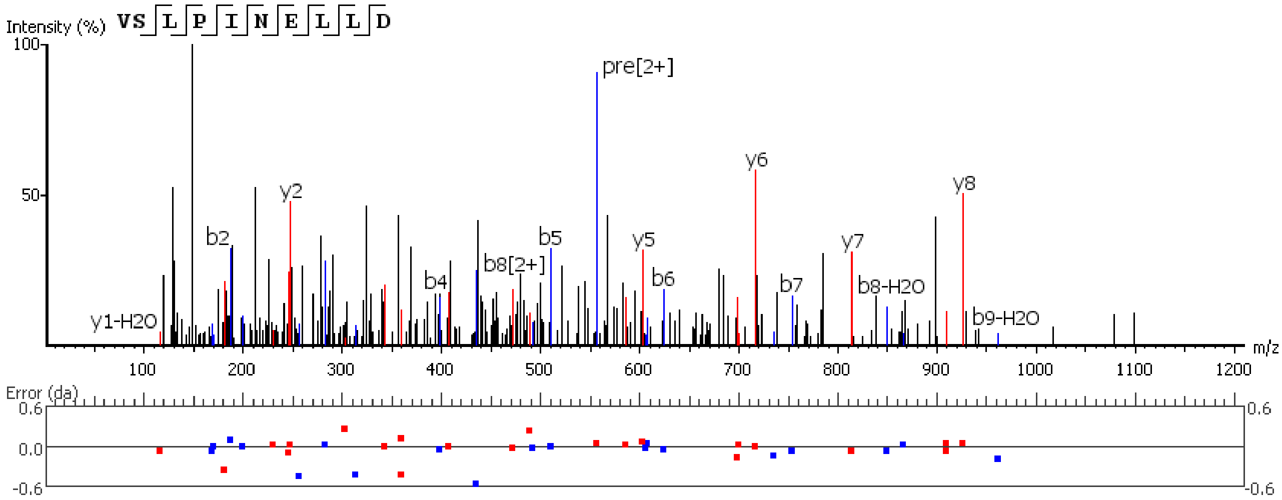

#	b	b-H2O	b-NH3	b (2+)	Seq	y	y-H2O	y-NH3	y (2+)	#
1	100.08	82.07	83.05	50.54	V					10
2	187.00	169.18	170.09	94.05	S	1013.55	995.54	996.52	507.28	9
3	300.19	282.18	283.12	150.60	L	926.46	908.60	909.43	463.76	8
4	397.31	379.23	380.22	199.10	P	813.53	795.42	796.41	407.20	7
5	510.34	492.35	493.30	256.13	I	716.38	698.55	699.32	359.12	6
6	624.43	606.39	607.30	313.11	N	603.22	585.26	586.27	301.88	5
7	753.49	735.55	736.39	377.21	E	489.00	471.24	472.26	245.24	4
8	866.47	848.58	849.47	434.33	L	360.08	342.22	343.19	180.97	3
9	979.58	961.76	962.56	490.29	L	247.09	229.12	230.06	124.06	2
10					D	134.04	116.12	117.02	67.52	1

Unknown

#17 FVCPLNLLAE

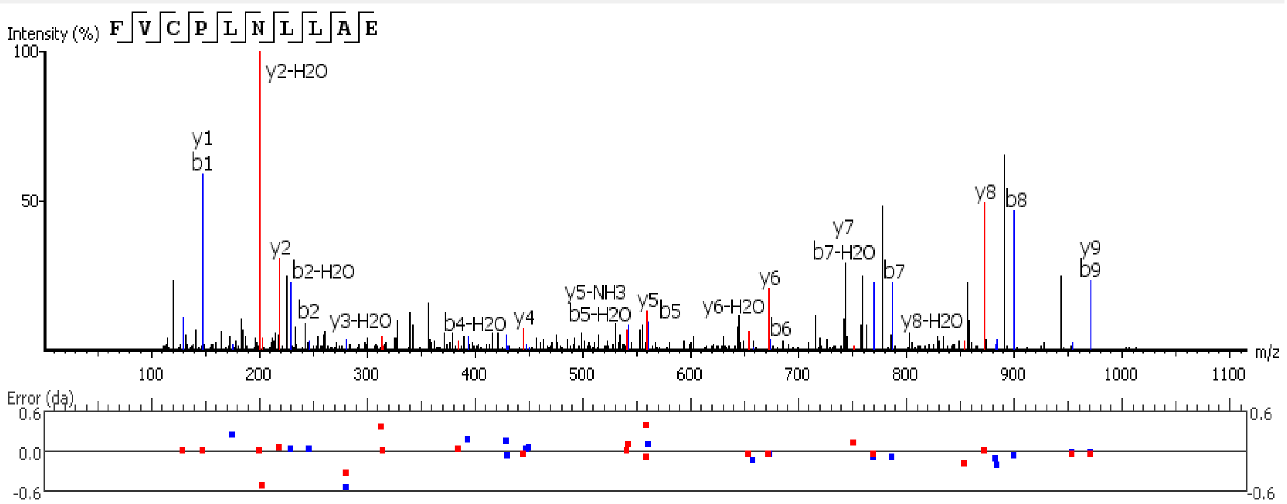

#	b	b-H2O	b-NH3	b (2+)	Seq	y	y-H2O	y-NH3	y (2+)	#
1	148.06	130.05	131.05	74.54	F					10
2	247.11	229.10	230.12	124.07	V	971.58	953.57	954.50	486.26	9
3	350.15	332.14	333.13	175.33	C	872.48	854.66	855.43	436.73	8
4	447.17	429.04	430.26	224.10	P	769.52	751.31	752.42	385.17	7
5	560.19	542.17	543.26	281.20	L	672.44	654.45	655.37	336.70	6
6	674.38	656.32	657.47	337.67	N	559.41	541.29	542.17	280.49	5
7	787.53	769.52	770.39	394.03	L	445.34	427.26	428.24	223.13	4
8	900.59	882.61	883.70	450.70	L	332.18	313.79	315.15	166.59	3
9	971.58	953.57	954.51	486.27	A	219.04	201.09	202.61	110.05	2
10					E	148.06	130.05	131.03	74.53	1
